# Supplementary material for: Novel multi-energy X-ray imaging methods: Experimental results of new image processing techniques to improve material separation in computed tomography and direct radiography
Source: PLoS One. 2020 May 6;15(5):e0232403. doi: 10.1371/journal.pone.0232403 (PMC7202619; doi:10.1371/journal.pone.0232403)
Supplement: Attachment — Submitted filename: rebuttal letter-2.docx [file pone.0232403.s005.docx]

Dear Reviewer,

Thank you very much for the examination of the paper and the constructive feedback. We hope that we addressed all the points raised. We will comment the changes on the following pages inside of your letter.

The authors introduce an empirical approach to perform a pixelwise fitting of the grey level of both

radiographic images and a series of volumetric images corresponding to a CT scan measured

with different X-ray tube voltages. The radiographs are fitted using a cubic interpolation while the

CT scans are fitted using an exponential function. I believe the results are very interesting and

depict a new method for empirical material differentiation on CT scans using conventional

hardware and multiple X-ray energies. Therefore, I suggest its publication after the changes

specified below. These are mostly required to justify the effectiveness of the approach, as well as

to improve the scientific and technical rigor of the paper.

Major points:

- I do not think the relationship between the proposed fitting and the X-ray imaging model

is clear. This mathematical relationship is, in my opinion, critical for replication of the

results in the case of other materials. Since the authors claim the method to be qualitative

rather than quantitative, my suggestion is for the authors to use a phantom with other

materials to show the empirical method can do material differentiation in other cases too

(Not as supplemental material with video 2 but as part of the discussion).

- We added to the manuscript results of another multi-energy CT scan where we investigated several minerals. There we used samples with similar mass density but different Z_eff_ and vice versa. Furthermore, we added a table with /rho and Z_eff_ as well as the fit parameters from the multi-energy CT-reconstruction. I hope that can clarify the empirical relationship between the mathematical model and the reconstructed X-ray data.

- Related to the previous comment, further discussion is needed on the selection of the

cubic interpolation for the radiography case. Furthermore, how did the authors get to the

conclusion on how to use \lambda, \gamma and \delta to generate the material images?

The relation with the physical quantities is not clear from the theory developed in the paper.

If the authors have explained this in a previous publication, please use a reference (line

125).

- The cubic interpolation and the use of the resulting fit parameters is completely empirically developed. We tried to clarify this in comparison to the numeric solution of Eq. (1) in line 112ff.

- The notation is confusing. The authors do a nice work getting to equation (3) but then they

go from V(t) to V(U) in equation (4) without an explanation and say it’s a derivation. This

needs to be explained. Furthermore, V(U) is supposed to be the measured intensity at

each tube voltage U, however, they claim to do a pixel/voxel grey level fitting for the CT

reconstruction using the expression V(U) too. The latter is very confusing since this gray

level corresponds to the attenuation coefficient quantities reconstructed for each voltage,

these are not intensities. Please change the notation or clarify this accordingly.

- The notation was unclear and partly wrong, we corrected that, thanks for mentioning this point. Furthermore for CT we changed V(U) to W(U) to clarify that it is the gray level after the CT reconstruction that we fit in that case. V(U) is than in all cases the measured gray level on the detector.

- Related to the previous comment, please explain why the second fit is on the linear

attenuation coefficients rather than on the intensities as derived in equation (4).

- We changed the whole paragraph and added a further equation (5). What we want so say is that we fit the intensities according to Eq. (1) with the approximation of Eq. (4) where we replace µ(iΔUe)t by the term containing the Klein-Nishina function.

- Seems to me from the manuscript that the user needs prior information of the materials

present in the sample to be able to identify which one corresponds to each parameter

fitted. Is this correct? If not, please clarify How can one know which material is in the

sample if the \rho \Z images are not quantitatively correct?

- Yes this is correct, that’s what we meant with material separation instead of material recognition. I tried to clarify this better now in the last paragraph of the results and discussion section

- I believe there is not enough detail on the fitting to replicate the results of the paper. The

authors do not give information on the type of software written, size of images, number of

voltages, size of the step in the tube voltage, selection of n, size of the sample. Among

others. Please elaborate, this information is often key for the reconstructions and cannot

be derived from the manuscript.

- We added some more information about the measurement parameters and the software. Furthermore, we uploaded the whole dataset used to get the results presented in the paper (scaled down in the case of CT due to size restrictions). Within the dataset, further measurement parameters are included. Additionally we added the source code to an updated version of the image analysis program on figshare. Following the private links to the data and updated program, it will all be made publicly available upon acceptance of the manuscript:

Data: https://figshare.com/s/a5fe112b8f67ee6a0dab

Program: https://figshare.com/s/596a87a9c3fd1d17d11e

Minor points:

- In the abstract the authors claim there are not stablished multi-energy methods. What

about photon counting detectors? The statement needs to be revised. Maybe clarify what

they mean with multi-energy method. On the other hand, to my knowledge there is one

paper that relies on photon counting detectors to attain \rho Z images [**] that is close a

multi-energy method. Please elaborate.

- We clarified that we mean multi-energy analysis with conventional hardware. We also cited the publication of Busi et. al.

- In equation (1), e (which I assume is the charge of the electron) is not defined.

- We defined it.

- In equation (4), K is not defined. Previous K in equation (2) came from the Kramer’s law

but with the simplification in equation (3), K is not present anymore. Is the K in equation

(4) different? Please, define.

- In Eq. (3) Kramers constant is not present because it is the relative intensity that is discussed there, we emphasized that. Furthermore K is now mentioned again just after the new Eq. (5).

- The link for the fitting program MultiE-GUI does not work. I was able to find a software with

the same name that seems to correspond to the description in the text, URL:

https://figshare.com/articles/MultiE-GUI_zip/9758390/1. Please fix this.

- We fixed the link.

- If I understand correctly the approach in reference [1] is not used to fit /mu due to the lack

of correction of the quantum efficiency at higher X-ray energies. Could you show the

results that you get when you try performing the fit with your data? From what I saw on

the .zip that I downloaded you have the option of performing this fit. I think this discussion

will help the readers see a clear motivation of the proposed approach.

- The results are now presented at the end of the multi-energy radiography section.

- There are some typos along the paper. Please revise carefully before resubmitting. Some

examples below:

o Line 34: that instead of than

o Line 112: change to: account for this behavior computationally

o Line 182: An image instead of a image

o Line 206: To an acceptable instead of to a acceptable

o Line 249: A more instead of an more

o Line 251: parameter instead of parameter

- The mentioned typos have been corrected, among others. I hope it’s better now.

[**] Busi, Matteo & Kehres, Jan & Khalil, Mohamad & Olsen, Ulrik. (2019). Effective atomic number

and electron density determination using spectral x-ray CT. 2. 10.1117/12.2519851.

Furthermore we changed the names of all fitting paramters to a more consistent scheme to avoid confusion.

Thanks again for revising the manuscript.

Kind Regards,

Mirko Heckert
